# Study on the Changing Law of Cutting and Ultrasonic Strengthening Surface Integrity during Fatigue of Ti-17 Alloy

**DOI:** 10.3390/ma15228106

**Published:** 2022-11-16

**Authors:** Zheng Zhou, Changfeng Yao, Liang Tan, Hongmin Xin, Ya Zhang, Yu Zhao

**Affiliations:** 1 Key Laboratory of High Performance Manufacturing for Aero Engine, Ministry of Industry and Information Technology, School of Mechanical Engineering, Northwestern Polytechnical University, Xi’an 710072, China; 2 Engineering Research Center of Advanced Manufacturing Technology for Aero Engine, Ministry of Education, School of Mechanical Engineering, Northwestern Polytechnical University, Xi’an 710072, China; 3 Aero Engine Corporation of China Shenyang Liming Aero-Engine Co., Ltd., Shenyang 110043, China; 4 Hubei Key Laboratory of Power System Design and Test for Electrical Vehicle, Hubei University of Arits and Science, Xiangyang 441053, China

**Keywords:** Ti-17, turning, ultrasonic impact, room temperature, surface integrity evolution

## Abstract

The distribution of surface integrity features directly affects the initiation and propagation of fatigue cracks. In this paper, the surface integrity characteristics changing law of turning and ultrasonic impacting specimens during high cycle fatigue loading has been studied, and the effect of surface modified layer on the fatigue properties of titanium alloy has been revealed. The results showed that the surface roughness increased with the increase of fatigue cycles. The compressive residual stress and its gradient distribution depth decreased continuously. The gradient distribution depth of residual stress in the ultrasonic-impacted surface rapidly decreased by about 50% near the fracture stage. Local cyclic hardening occurred at 20–50 μm from the surface of the specimen in the early stage of fatigue evolution, and then the microhardness continued to decrease. During this process, there were no significant changes in hardened layer depth. The fibrous microstructure of the ultrasonic-impacted surface undergoes a process from coarsening to gradual disintegration during the fatigue process. Its attenuation process needs a longer period of time. The fatigue source of the turned specimen was located at about 320 μm from the surface, and the fatigue source of ultrasonic impact was about 610 μm from the surface. The fatigue striation width of the ultrasonic impact specimen was about 20% narrower than that of the turned specimen. The fatigue life of the ultrasonic impact specimen was increased by 73.9% compared with the turned specimen. The research in this paper is of great significance for exploring the anti-fatigue mechanism and the ability of various surface integrity features.

## 1. Introduction

Fatigue performance is an important indicator for evaluating the quality of parts [1,2]. More than 80% of the failures of mechanical parts are attributable to fatigue failure. The fatigue failure of metal materials is the result of the accumulation of material damage and the evolution of surface integrity [3,4]. Surface integrity is an important basis for studying the accumulation of material damage in the fatigue process as a relatively scientific and complete characterization system of the material’s machined surface. Compressive residual stress can inhibit the initiation of microcracks and hinder crack propagation. The gradient hardening layer can increase the threshold of crack initiation and propagation, thereby affecting the crack growth rate [5].

With the continuous action of the alternating load, “intrusion” and “extrusion” grooves are more likely to be formed on the surface of the plastic material. This results in increased surface roughness and microscopic stress concentrations. The surface roughness characterization parameters *R_a_*, *R_q_*, *R_p_*, and *R_v_*, were used to study the fatigue damage of the surface [6]. The residual stress relaxed or redistributed under cyclic loading. The relaxation of residual stress during fatigue has a strong correlation with the development of plastic strain amplitudes. The relaxation process of residual stress can be divided into two stages: first, the macroscopic stress in the near-surface region disappears, and then, the microstructure evolution in the near-surface region reduces the residual stress [7].

Han S et al. [8] studied the residual stress relaxation characteristics in the steel welding area and proposed a model to quantitatively predict the residual stress relaxation amount. Relaxation occurs when the sum of the local external load and residual stress exceeds the yield stress of the material. The residual stress is significantly relieved under the first cyclic loading and then gradually relaxes with the increase of cycles. Zaroog et al. [9] studied the stability of residual stress of 2024-T351 aluminum alloys with different shot peening strengths under cyclic loading conditions. The relaxation of residual stress was found to be somewhat dependent on the load amplitude. The residual stress relaxation becomes more obvious with the increase of the load amplitude [10,11]. In the first 10 cycles, the residual stress relief was significant. From 10 to 10,000 cycles, the relaxation percent of all specimens varied within 5–8% of the residual stress in the first cycle. During the first loading cycle, the relaxation of residual stress is the result of the quasi-static relaxation effect. In subsequent cycles, the residual stress is gradually relaxed, and the residual stress relaxation has a logarithmic relationship with the number of cycles.

The shot peening residual stress in 7075-T651 aluminum alloy sheet relaxes significantly in the first cycle and then tends to be stable. The residual stress has been in a stable state under the condition of low load [12]. In addition, the stress ratio *R* and temperature also have important influences on the relaxation of residual stress. When the R-value was 0.1, the residual surface stress did not decrease significantly. When the R ratio was −1, the residual stress decreased significantly in the first cycle was then maintained at approximately 150 MPa until the end of the cyclic load [13].

The surface rolling strengthening the residual stress gradient distribution layer in Ti-6Al-4V and IMI679 materials is affected by temperature. Under room temperature and low cycle fatigue conditions, the residual stress relaxation degree is less than 50% of the peak residual stress. In comparison, the maximum residual stress relaxation degree can reach 70% of the peak value of the residual stress at high temperatures [14].

For austenitic steel AISI304, the amount of residual stress relaxation mainly depends on the temperature. When the temperature is in the range from room temperature to 200 °C, which depends on the stress amplitude when the temperature is between 200 and 600 °C. The higher the temperature, the earlier the residual stress relaxed [15]. The microhardness and microstructure of the material also change accordingly with the relaxation of residual stress. An increase in mechanical load leads to an increase in the drop amount of microhardness [9]. The change of microhardness is disordered when the number of cycles is small and then increases with the increase of the number of cycles [11]. The dynamic recrystallization in AZ31B magnesium alloy during tension-stretch cyclic loading results in a rapid reduction of equiaxed grains at 5000 cycles [16]. When the cyclic stress is large, a kind of cell-like structure is produced in the SAE 1045 shot-peened metamorphic layer due to the change of dislocation arrangement [7]. The metal in the weld area of 304L stainless steel and ER308L exhibits a brief hardening phase in the first few cycles under axial strain cyclic loading, followed by cyclic softening until cracking, with a gradual decrease in dislocation density [17]. The nanograins in the ultrasonic rolled surface layer of Ti-6Al-4V coarsen under uniaxial loading. Increasing the strain amplitude results in an increase in the growth rate of the nanograins. The growth of nanograins is driven by strain energy, which is related to the relaxation of residual stress. The grain growth rate can be approximately expressed as an exponential function of the strain amplitude [18].

Scholars have also conducted a lot of research on the damage to materials in the fatigue process. Material damping parameters are used to evaluate the behavior of fatigue evolution. The evolution of damping parameters goes through three stages: the initial stage, the steady state stage, and the abrupt stage before final failure. Crack initiation and fatigue life can be predicted according to the change of damping [19]. J. Kumar et al. performed numerical simulations of fatigue damage of α-titanium alloy specimens using finite element analysis. They analyzed the damage evolution in low-cycle high-temperature fatigue tests [20]. A. A. Griffith first proposed the theory of solid crack propagation in 1921 [21]. In 1945, Miner proposed the fatigue linear cumulative damage evolution theory, which solved the problem of fatigue life prediction under variable amplitude loads [22]. In 1963, P. C. Paris deduced the law of crack propagation-Paris formula by the method of fracture mechanics. This provided a method for estimating the crack propagation life [23]. Now, the study of the fatigue problem extends from deterministic fatigue to probabilistic fatigue, through the estimation of fatigue life to the study of the evolution behavior of the whole fatigue process [24].

Existing research mainly focuses on the evolution of the microstructure; there are few studies on the evolution or changing law of surface integrity characteristics. In this paper, the changing laws of surface roughness, surface morphology, microhardness, residual stress, and microstructure in the surface layer during the fatigue process of turning and ultrasonic impact specimens were compared and analyzed. The fatigue fracture, fatigue strip, and fatigue crack propagation under the two processes have been observed and analyzed. The mechanism of surface integrity features action during the fatigue process has been revealed.

## 2. Material and Experiments

### 2.1. Materials and Fatigue Specimen Preparation

The titanium alloy material used in this study was Ti-17. Its nominal composition is Ti-5Al-2Sn-2Zr-4Mo-4Cr. It is an α-β type two-phase titanium alloy and rich in α-stabilizing elements with a maximum working temperature of 427 °C. It has a series of advantages, such as high strength and good fracture toughness. The main chemical compositions of Ti-17 are listed in Table 1, and the mechanical properties are listed in Table 2.

**Figure 1:**
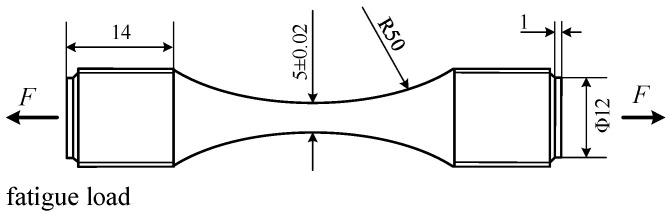
Fatigue evolution specimen (unit: mm).

The specific size of the sample is shown in Figure 1. There were threads at both ends to facilitate the clamping of the fatigue tester.

**Figure 2:**
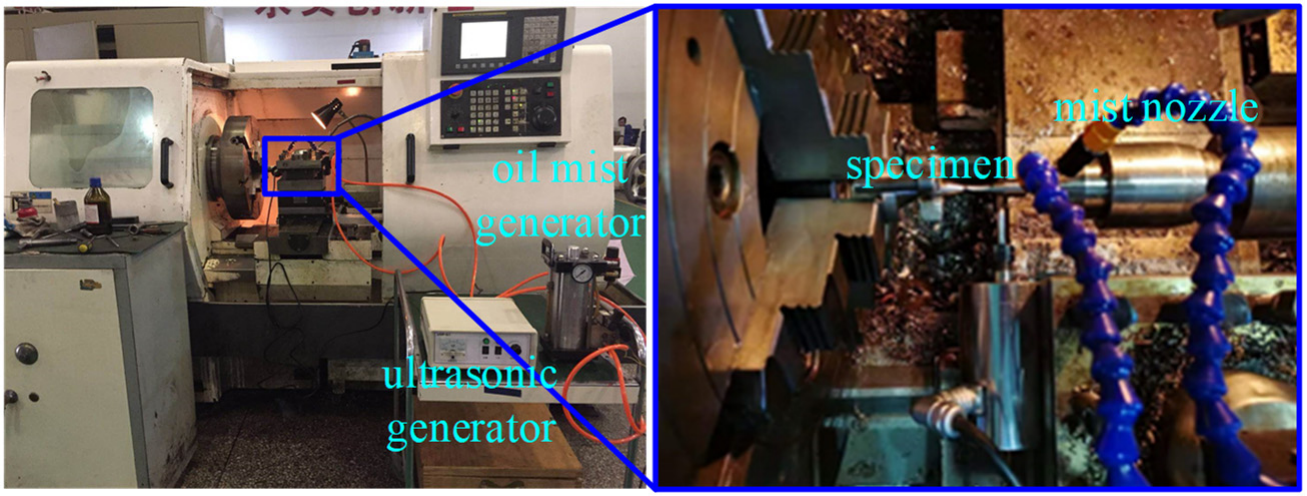
Ultrasonic impact process.

The geometric size of the fatigue specimen is guaranteed by the turning process, and the subsequent ultrasonic impact strengthening process was carried out on the basis of the turning surface. The turning parameters of the fatigue sample were: the cutting depth *a_p_* = 0.2 mm, the feed rate *f* = 0.06 mm/r, and the rotating speed *n* = 300 r/min. The ultrasonic impact parameters were: impact depth was 0.01 mm, the feed rate was 0.04 mm/r, and rotating speed was 50 r/min. The impact depth is the depth of the impact head pressed into the material surface, and the static pressure during the ultrasonic impact process was provided by impact depth. The ultrasonic frequency was about 34 kHz, and the diameter of the impact head was 4 mm. Oil mist lubrication was adopted during the ultrasonic impact process. The ultrasonic impact experiment is shown in Figure 2.

### 2.2. Fatigue Test

**Figure 3:**
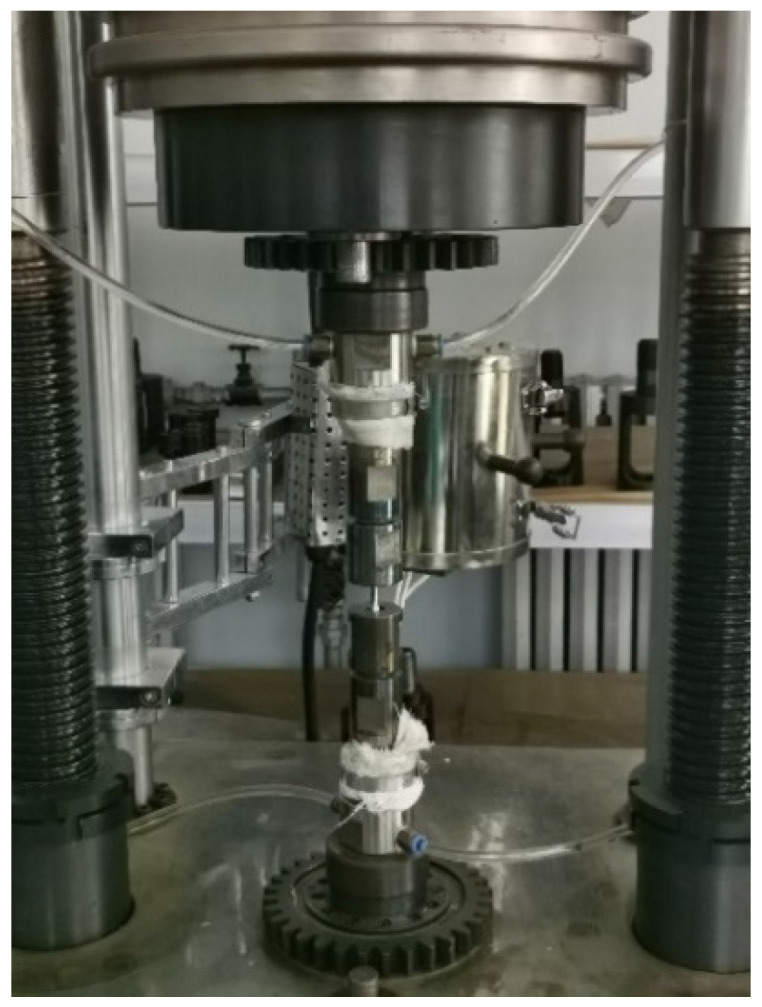
QBG-50 High-frequency fatigue tester.

A QBG-50 high-frequency fatigue testing machine was used in this experiment. Its maximum static load is in the range of ± 50 kN, and the maximum dynamic load was 25 kN. Figure 3 shows the fatigue testing machine. The stress ratio *R* was set to 0.1 with no compressive stress load. The cyclic load was applied in the form of a sine wave. This experiment was carried out at a room temperature of 20 °C. The loading frequency was 115 Hz, and the error range was within ± 3%. In the fatigue evolution experiment, different fatigue cycles *N*_1_, *N*_2_, *N*_3_, and *N*_4_ were determined according to the fatigue life *N_f_* of the specimen. The surface integrity of the specimens in these four stages of fatigue evolution was tested. In order to make the results accurate and reliable and improve the test efficiency so that the fatigue life will not overflow, it is necessary to set the fatigue load so that the cycles of fatigue fracture are optimal below 10^6^. Three specimens were used for the load selection test, and the load was set to 820 MPa finally. 

Fatigue specimens were divided into turning specimens (T_1_, T_2_, T_3_, T_4_) and ultrasonic impact specimens (U_1_, U_2_, U_3_, U_4_, U_5_). T_4_ was a fatigue fracture specimen whose fracture cycle was *N*_T4_ = *Nf*_T_ = 9.66 × 10^5^ cycles. T_2_ and T_3_ were in the intermediate process of fatigue evolution. The fatigue cycles were *N*_T2_ = 10^−2^ *Nf*_T_ = 9.66 × 10^3^ and *N*_T3_ = 10^−1^ *Nf*_T_ = 9.66 × 10^4^. T_1_ was a non-fatigue cycle specimen; its cycle time was *N*_T1_ = 0. The ultrasonic impact specimen U_5_ was a fatigue fracture specimen, and its fracture cycle was defined as *N*_U5_ = *Nf*_U_ = 1.68 × 10^6^ cycles. U_2_ and U_3_ were in the intermediate process of fatigue evolution. The fatigue cycles were *N*_U2_ = 10^−2^ *Nf*_U_ = 1.68 × 10^4^ cycles and *N*_U3_ = 10^−1^ *Nf*_U_ =1.68 × 10^5^ cycles, respectively. The cycle number of U4 was *N*_U4_ = *Nf*_c_ = 9.66 × 10^5^, and the U_1_ was *N*_U1_ = 0.

### 2.3. Surface Integrity Test

XT20 surface profiler was used to test the surface morphology and surface roughness of the specimens. The surface morphology test area was 1 mm^2^, and the roughness value was tested along the axial direction of the sample. The sampling length was set to 0.8 mm, and the evaluation length was 4 mm. Each sample was measured at intervals of 72° in the direction of the circumference 5 times. The average value of surface roughness was taken as its final surface roughness. The surface microstructures were observed using a Helios G4 CX scanning electron microscope with a magnification range of 500 to 8000. The microhardness gradient distribution was tested using a FEM-8000 Vickers microhardness tester with a test load of 20 gf and load time of 10 s. The distance between adjacent test points was 10 µm in the depth direction and 40 µm in the horizontal direction. The residual stress was tested by PROTO-LXRD MG2000 X-ray residual stress test system, produced by the Canadian company PROTO. The residual stress gradient distribution was measured by immersion method in corrosion solution. An acid solution (HF/HNO_3_/H_2_O = 1:5:14 by volume) was used for the corrosion of microstructure samples [25]. Fatigue fractures and microstructures were tested using an Oxford scanning electron microscope instrument.

## 3. Results and Discussion

### 3.1. Evolution of Surface Characteristics

**Figure 4:**
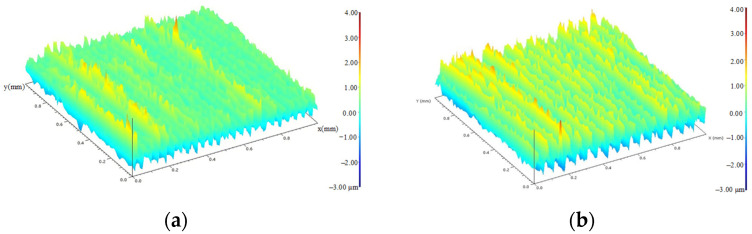

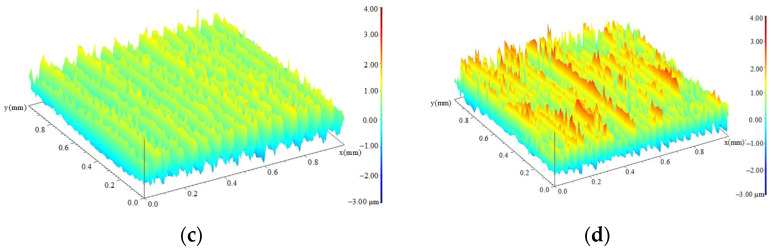
Surface topography of turned specimens during fatigue evolution. (**a**) 0 cycles; (**b**) 9.66 × 10^3^ cycles; (**c**) 9.66 × 10^4^ cycles; (**d**) 9.66 × 10^5^ cycles.

Figure 4 shows the surface morphology of the turned specimen after different cycles. They are typical turning characteristics with almost parallel grains, which are determined by the feed rate and the cutting depth. The direction in which the fatigue load was applied was almost perpendicular to the direction of the surface texture. The distance between adjacent peaks or valleys on the surface was relatively small, and the surface was relatively flat. The heights of the contour peaks were basically the same. Figure 4b,c shows the surface morphology of the specimen during fatigue cycles. It can be seen that the increase of profile peak height shows a gradual process from part of the region to the whole surface. Figure 4d shows the surface morphology near the fracture region of the fractured specimen. The uneven distribution of the red area indicated that the peak height of the local contour was further increased at this stage, and the surface roughness was also significantly increased. The increase of surface slip band during fatigue was the main reason for the continuous deterioration of surface quality.

**Figure 5:**
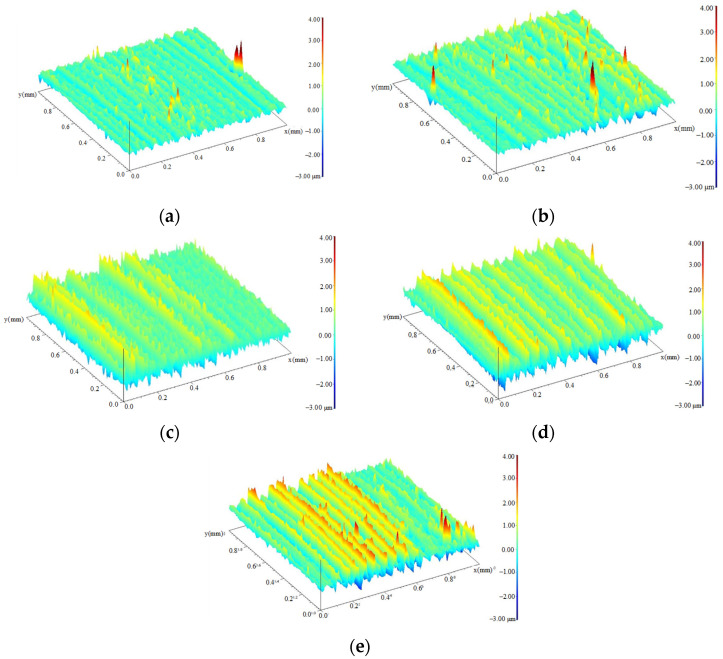
Surface topography of ultrasonic-impacted specimens during fatigue evolution. (**a**) 0 cycles; (**b**) 1.68 × 10^4^ cycles; (**c**) 1.68 × 10^5^ cycles; (**d**) 9.66 × 10^5^ cycles; (**e**) 1.68 × 10^6^ cycles.

Figure 5 shows the surface morphology of the ultrasonic-impacted specimens in different cycles. Figure 5a shows the surface morphology of the specimen with a fatigue cycle of 0. The surface is relatively flat. Figure 5b–d shows the surface morphology of the specimen during the fatigue process. Similar to the turned surfaces, the surface unevenness and the distribution areas of the yellow and red contour peaks gradually increased with the increase of cycles. It can be seen that the change in the surface quality was a periodic process. The deterioration of the surface quality was from a local area, then expanded to a larger area. As a complete cycle of surface morphology changed, this process occurred back and forth with the cyclic loads. Figure 5e is the fracture specimen. The surface contour peaks were arranged irregularly, and the surface quality was seriously deteriorated. 

**Figure 6:**
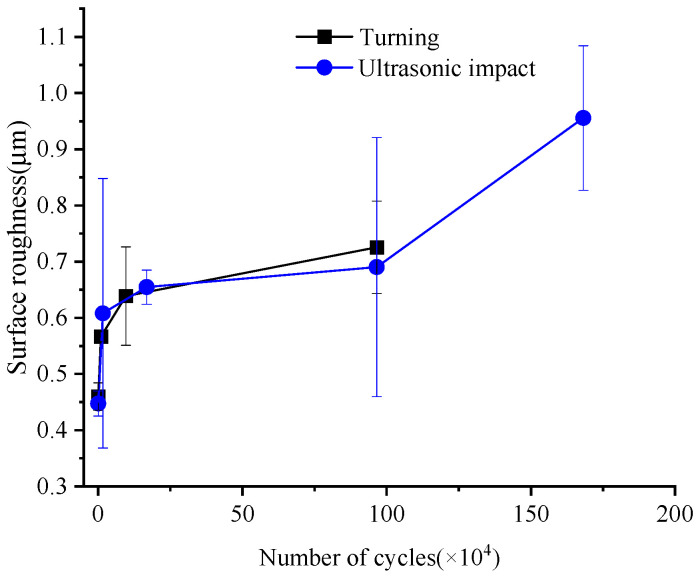
Surface roughness Ra changes during fatigue evolution.

Figure 6 shows the surface roughness changes during the fatigue process. The roughness height parameters *R_a_* of the turning and ultrasonic impact specimens showed a monotonous increasing trend, and the surface became rougher and rougher until the specimen fractured. In the initial stage of the fatigue process, within about 10,000 cycles, the roughness increased sharply. From 10,000 cycles to fracture, the increasing rate of roughness slowed down, and the final increased from 0.46 μm to 0.73 μm, which increased by 57.6%. Similar to the evolution of the surface roughness of the turning specimen, the rate of the ultrasonic impact specimen increased sharply at the beginning of the fatigue process and then decreased, showing an obvious “platform period.” From 960,000 cycles to the point where the fracture occurred in the specimen, the increase rate of roughness was further accelerated and showed a sharp increase trend. Finally, the roughness value increased to 0.955 μm with an increase of 113.6%.

### 3.2. Evolution of Residual Stress Distribution

**Figure 7:**
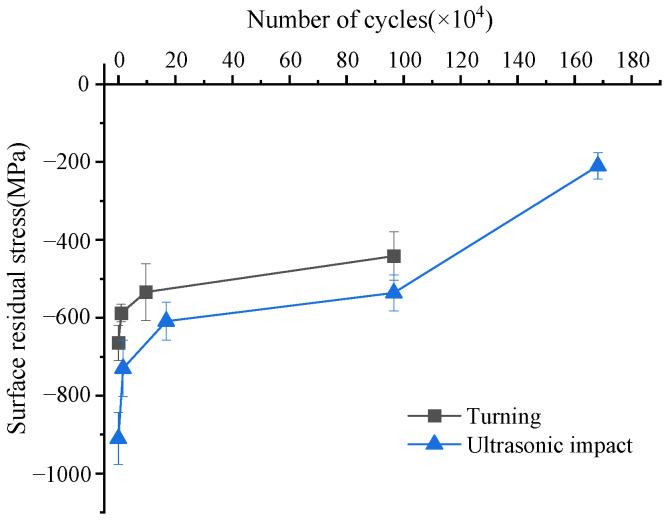
Variation of residual surface stress during fatigue.

Figure 7 shows the changes in residual surface stress during fatigue cycles. Both turned and ultrasonic-impacted initial surfaces exhibited compressive residual stress states. With the increase in fatigue cycles, the surface compressive residual stresses gradually decreased. The initial surface compressive residual stress of the turned specimen without fatigue cycles was about 670 MPa. After about 10,000 fatigue cycles, the surface compressive residual stress dropped sharply to about 580 MPa. During the fatigue process, the cyclic loads on both ends of the specimen were opposite to the residual stress so the compressive residual stress was released to a certain extent. When the specimen broke, the residual compressive stress on the surface dropped to about 440 MPa. In the initial stage of fatigue, the surface compressive residual stress was released rapidly. With the increase in fatigue cycles, the release rate of compressive residual stress gradually slowed down. The surface material of the ultrasonic impact specimen underwent a large plastic deformation during the processing, and large compressive residual stress was formed in the surface layer. The compressive residual stress on the surface of the specimen without fatigue cycle was about 900 MPa. After 16,000 cycles, the surface compressive residual stress dropped sharply to about 730 MPa. Between 16,000 and 960,000 cycles, the decay rate of the surface compressive residual stress gradually slowed down. After 960,000 cycles, the surface compressive residual stress was about 530 MPa. The specimen fractured after 1.68 million cycles, and the final surface compressive residual stress was about 210 MPa. Compressive residual stress can effectively improve fatigue life—the greater the compressive stress, the greater the improvement of fatigue performance. The compressive residual stress reduces the actual load of the material during the fatigue cycle [26]. On the one hand, the threshold for the fatigue crack initiation is increased, and the formation of microcracks is delayed. On the other hand, microcracks cannot open and propagate normally under high compressive stress; the propagation process of cracks is delayed.

**Figure 8:**
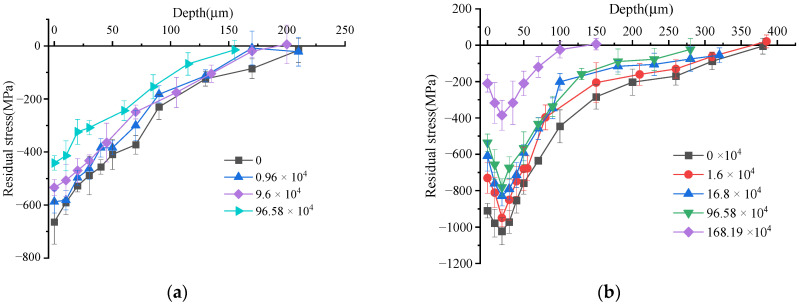
Residual stress distribution evolution during fatigue process. (**a**) Turning residual stress; (**b**) Ultrasonic impact residual stress.

Figure 8 shows the distribution of residual stress gradient in the surface layer during fatigue cycles. It can be seen from Figure 8a that the residual stress of the turned samples was all compressive stress. The maximum compressive residual stress appeared on the material surface and gradually decreased along the depth direction. The depth of the residual stress gradient distribution layer was about 170–200 μm. The compressive residual stress on the surface of the specimen without fatigue cycle was the largest, and its gradient distribution depth was more than 200 μm. After about 10,000 cycles, the residual surface stress decreased from 664 MPa to 587 MPa, and its gradient distribution depth decreased by about 24 μm. After 96,000 cycles, the surface compressive residual stress decreased to about 533 MPa, and its gradient distribution depth decreased slightly. After the specimen experienced 96,000 fatigue cycles until its fracture, the surface compressive residual stress underwent a sharp decrease. The residual stress at each position was reduced by about 50–100 MPa, and the depth of the gradient distribution layer was reduced by about 20–30 μm. In general, the compressive residual stress value and the depth of the gradient distribution layer decreased with the increase of the fatigue cycles during the fatigue process of the turned Ti-17specimen. In the initial stage of fatigue cycles, the compressive residual stress and its gradient depth decreased slowly. When the fatigue specimen approached the fracture stage, the compressive residual stress and the gradient distribution layer decreased significantly, with a large amount of residual stress being released.

It can be seen from Figure 8b that the residual stress of ultrasonic impact samples all showed compressive stress and showed in the form of a “leaky spoon,” obviously. Theoretically, the surface layer of the sample is in a state of compressive residual stress after ultrasonic impact. The specimen surface is a free surface. On the one hand, the residual surface stress has a tendency to release. On the other hand, the strain on the surface is constrained by the internal material. Therefore, the surface residual compressive stress can only be partially released. This resulted in a “leaky spoon” shaped distribution curve with the maximum residual stress at the subsurface. The compressive residual stress was greater than that of the turned specimen. The compressive residual stress gradually increased from the surface to its peak value in the depth direction. The maximum compressive residual stress appeared at about 20 μm from the surface layer and then gradually decreased until it was equal to the matrix residual stress.

It can be seen that the compressive residual stress and its gradient layer depth were the largest before fatigue cycling. The surface compressive residual stress was about 910 MPa, and its peak value was 1023 MPa. Its gradient distribution layer depth was about 380 μm. After 16,000 fatigue cycles, the compressive residual stress decreased. Its peak value was 949 MPa, and the gradient distribution layer depth was about 370 μm. After 168,000 fatigue cycles, the compressive residual stress dropped more obviously, with a peak value of 828 MPa. The gradient distribution depth was about 350 μm. After 960,000 fatigue cycles, the peak value of the residual compressive stress was 781 MPa, and its gradient distribution depth dropped sharply to about 280 μm. The specimen fractured after 1.68 million fatigue cycles. The residual stress at the same position dropped by about 300 MPa compared with when it was 960,000 cycles, and the peak value of residual stress was only 385 MPa. At the same time, the gradient distribution depth also dropped sharply to about 130 μm. During the fatigue process of the ultrasonic-impacted specimen, the compressive residual stress values and the gradient distribution depth were all decreased. Similar to the evolution law of turning residual stress, the decay rate of residual stress and its gradient distribution depth experienced a gradually increasing process.

### 3.3. Evolution of Microhardness Distribution

**Figure 9:**
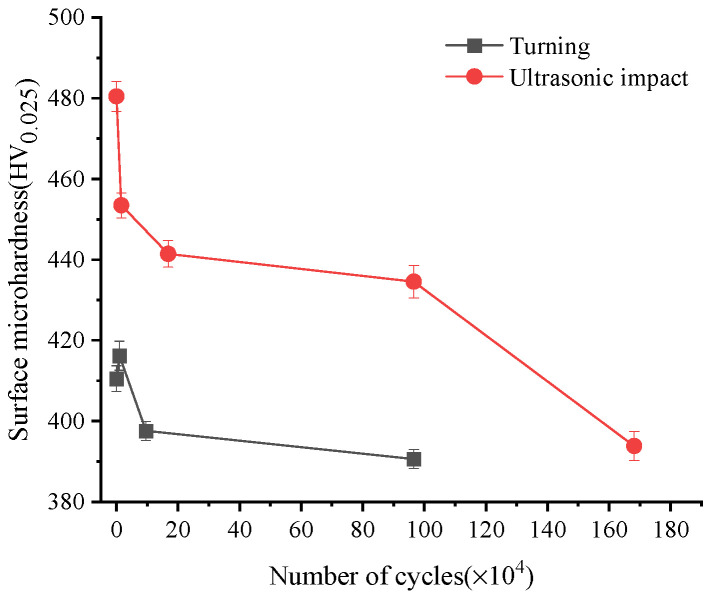
Surface microhardness evolution during the fatigue process.

In the process of fatigue evolution, the cyclic plastic deformation of the specimen is also one of the reasons for the fatigue damage accumulation. Cyclic plastic deformation shows the characteristics of cyclic hardening and softening in the material’s mechanical properties, which can be characterized by microhardness. Figure 9 shows the surface microhardness changes of Ti-17 titanium alloy specimens during fatigue cycles. The surface hardness of turned specimen was about 410 HV when the number of cycles was 0. After about 10,000 fatigue cycles, the surface hardness of the specimen increased, which was manifested as cyclic hardening. After that, the surface hardness dropped sharply until it broke and showed the characteristics of surface softening. Its surface hardness was about 390 HV finally. In the early stage of fatigue, some surface grains were in individual orientations were easy to slip first and undergo cyclic slip and hardening. The plastic deformation resistance and surface hardness of the material increased in this process. With the increase of fatigue loading cycles, the cyclic plastic deformation of the specimen increased, which leads to the softening of the material.

The surface hardness value of the ultrasonic-impacted specimens was the largest when the cycle number was 0, which was about 480 HV. After about 10,000 fatigue cycles, the surface hardness decreased to about 453 HV sharply. After that, the surface hardness decreased slowly and entered an obvious plateau stage. Then it dropped sharply until it broke. The surface hardness was about 390 HV in the end. The surface and sub-surface grains were broken into small grains under a large mechanical load after the specimen surface had been strengthened by ultrasonic impact. The dislocations in surface material increased sharply, and the plastic deformation resistance was also improved. The surface hardness of the material has reached the peak value, so that the surface hardness of the specimen without cycle was the largest. With the increase of cycles, the plastic slippage of the surface grains was hindered, and the dislocation configuration rapidly evolved to the saturated dislocation structure (cellular structure). This kind of cellular structure represented a low-energy state compared with the original dislocation configuration and manifested by a sharp increase in fatigue damage and a sharp decrease in surface hardness. In the subsequent fatigue cycle process, it was more manifested as the expansion process of the fatigue crack tip, and the surface hardness decreased slowly. In the near-fracture stage, the crack structure in the material mainly developed and extended in some local area. The crack expansion and merger made the surface material further soften. The surface hardness dropped sharply again when the specimen broke.

**Figure 10:**
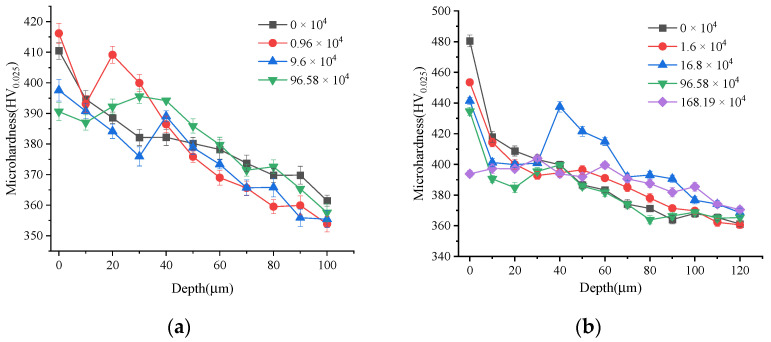
Variation of microhardness gradient distribution during fatigue. (**a**) Turned surface; (**b**) Ultrasonic-impacted surface.

Figure 10 shows the gradient distribution of microhardness during fatigue cycles. From Figure 10a, it can be seen that the microhardness of the surface layer of the turned sample gradually decreased along the depth direction and showed a softening effect as a whole. The hardened layer depth was about 100 μm, and the material matrix hardness was about 350–360 HV. The surface microhardness of the specimens without cycling decreased gradually along the depth direction until the matrix hardness. After about 10,000 fatigue cycles, the hardness value of the specimen surface layer increased significantly at a depth range of 10–30 μm from the surface and showed a hardening effect. Then the microhardness gradually decreased after 20–30 μm. In the process of fatigue loading, the dislocation density increased, and the dislocation moved to the depth direction of the surface layer. Some areas in the surface layer showed strain-hardening states. After 100,000 fatigue cycles, the microhardness of the material in the range of 30 μm from the surface decreased sharply but increased significantly at about 40 μm from the surface. During the fatigue process, the expansion of the crack tip needs to dissipate energy. The increase of the material damage degree in the fatigue process led to a sharp decrease in microhardness. The surface hardness of the turned specimen T_2_ and specimen T_3_ indicated that the microcracks continued to expand and grow along the depth direction. The plastic deformation of the crack tip caused the strain hardening of the material. The specimen T_4_ was the fractured part, and its fatigue life was 960,000 times. It can be seen that its surface hardness was the lowest and was similar to that of T_3_. This indicated that the softening effect of surface hardness decreased gradually when the cycles were close to the fatigue life.

Figure 10b shows the microhardness gradient distribution change of the ultrasonic impact specimen during the fatigue process. The hardened layer depth of the ultrasonic impact specimen was about 120 μm. The initial surface microhardness and gradient layer depth were about 482 HV and 90 μm. After nearly 10,000 fatigue cycles, the surface hardness changed little, while appeared the surface softening effect. The microhardness decreased slowly from 30–50 μm from the surface. After 168,000 cycles, the microhardness decreased sharply in the range of 0–20 μm from the surface but increased significantly in the range of 30–50 μm. The specimen went through the initiation stage of micro-cracks, and local cyclic deformation occurred in the material near the crack tip. This resulted in strain hardening of the material and the increase of microhardness at this position. After 966,000 fatigue cycles, the hardness continued to decrease in the range of 0–20 μm from the surface but increased in the range of 20–50 μm. Local hardened occurred in the material near the surface, while a softened state appeared in the whole surface layer. After 1.68 million fatigue cycles, the specimen fractured. The surface hardness dropped to the lowest level with a partial increase in the range of 20–40 μm from the surface and a decrease in the range of below 60 μm. The material showed a softening effect. In the stage of rapid expansion of the fatigue crack, the softening effect gradually decreased.

### 3.4. Evolution of Microstructure

**Figure 11:**
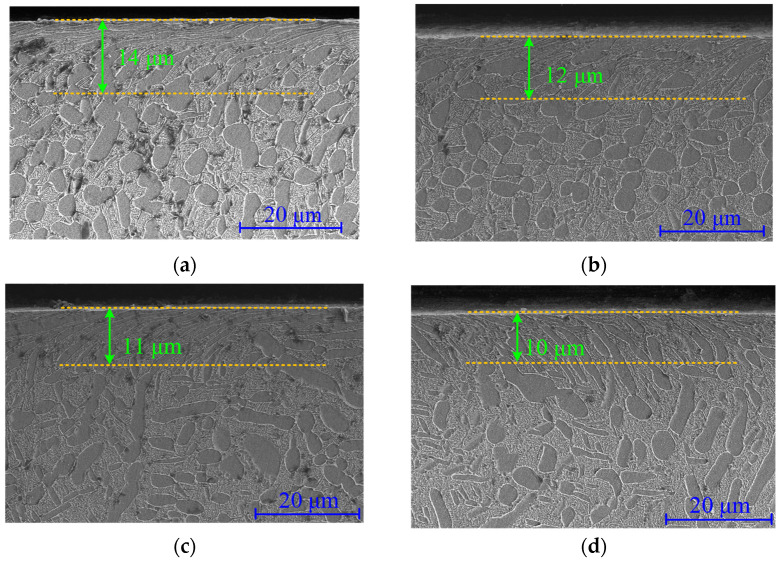
Microstructure characteristics of turned surface during fatigue process. (**a**) 0 cycle; (**b**) 9.66 × 10^3^ cycles; (**c**) 9.66 × 10^4^ cycles; (**d**) 9.66 × 10^5^ cycles.

**Figure 12:**
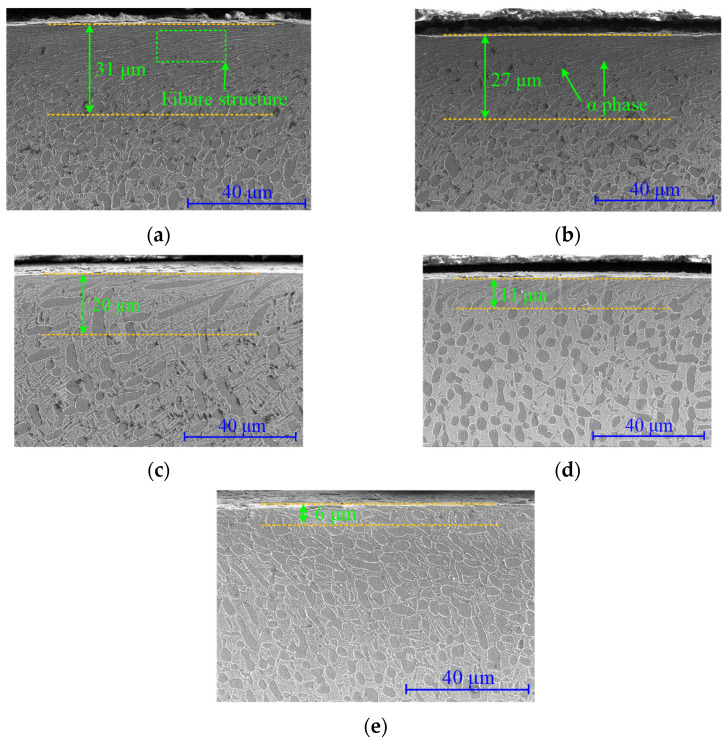
Microstructure characteristics of ultrasonic-impacted surface during fatigue process. (**a**) 0 cycle; (**b**) 1.68 × 10^4^ cycles; (**c**) 1.68 × 10^5^ cycles; (**d**) 9.66 × 10^5^ cycles; (**e**) 1.68 × 10^6^ cycles.

Figure 11 and Figure 12 show the material microstructural features evolution during the fatigue process for turning and ultrasonic impacting the surface. It can be seen that certain depths of plastic deformation layers were formed on the surface after turning and ultrasonic impact. The α phase in the material was bent and deformed, elongated, and flattened along the line speed direction. The grain size was distributed in a gradient along the depth direction. The depth of the ultrasonic impact surface deformation layer was larger. The grain boundaries in the surface layer became blurred and difficult to distinguish and formed a kind of dense fibrous layer. The streamlines formed by plastic deformation have a smaller angle with the material surface compared with that in the turned surface. About 14 μm of the plastically deformed layer was formed in the turning surface, while the thickness of the plastically deformed layer in the ultrasonically impacted surface was about 31 μm. 

The depth of the turning deformation layer slowly decreased to 10 μm with the increase of cycle numbers. During this process, the bending degree of the α phase decreased. The width of the α phase and the spacing between the phase boundaries gradually increased. The thickness of the plastic deformation layer on the ultrasonic impact surface decreased from 31 μm to about 6 μm when fractured. With the increase of the fatigue cycle numbers, the fibrous morphology in the surface layer gradually changed. The spacing of the fibrous gradually increased, and some scattered long strips of α phase appeared. The increasing number of elongated α-phase gradually disintegrated the fibrous structural layer, while the plastic deformation layer depth decreased significantly. The ultrasonic impact process greatly increased the dislocation density in the surface material. It enhanced the plastic deformation resistance of the material, thereby forming a deep compressive residual stress gradient layer and hardened gradient layer. During the fatigue process, the changes in the plastic deformation layer depth and its morphology were directly reflected in the attenuation of compressive residual stress and microhardness. Compared with the turned surface layer, the attenuation process of the significant plastic deformation and the grain refinement in the ultrasonic-impacted surface layer need a longer period of time, which is of great significance to improve the fatigue performance of the material.

### 3.5. Fatigue Fracture Analysis

In order to deeply analyze the surface integrity evolution of turned and ultrasonic-impacted specimens during the fatigue process, an SEM test has been carried out to observe the fatigue fracture zone. The fatigue source zone, fatigue propagation zone, and transient break zone were investigated.

**Figure 13:**
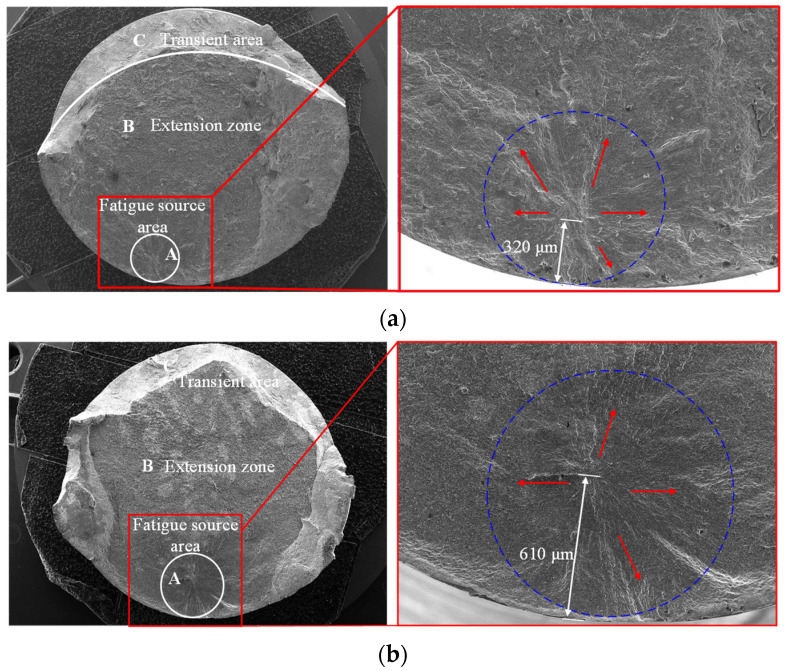
Fatigue fracture specimen. (**a**) Turned specimen; (**b**) Ultrasonic-impacted specimen.

Figure 13 shows the macroscopic morphology of the fatigue fracture of the specimen. The fatigue life of the turned specimen was 966,000 cycles. The morphology of the fatigue source area was relatively flat and smooth and had obvious cleavage surface characteristics. The fracture type belonged to the single source initiation, and the initiation position of the crack was about 320 μm from the material surface. The cracks spread to the surroundings along radial kind paths, with obvious fatigue step characteristics. Figure 13b shows the fatigue fracture diagram of the ultrasonic-impacted specimen. Its fatigue life was 1.688 million cycles. The fatigue source area was flat and smooth. It was darker in color and had a round shape. The fracture type also belonged to the single source initiation, and the origin of the crack was about 610 μm from the material surface. Compared with the turned sample, the fatigue source was located at a deeper position from the specimen surface. This is because the ultrasonic impact strengthening process refined the grains in the surface layer and increased the number of grain boundaries of the surface layer. When the fatigue crack propagated through the grain boundary, the resistance from the grain boundary was increased due to the different grain orientations on both sides of the propagation path (the grain boundary). At the same time, the high dislocation density in the ultrasonic impact-strengthened layer and the blocking effect from the new grain boundaries increased the anti-slip ability of the grains in the strengthened surface layer, so it was difficult for microcracks to initiate in the impact-strengthened surface layer. This resulted in the crack source appearing at a deeper position from the surface.

**Figure 14:**
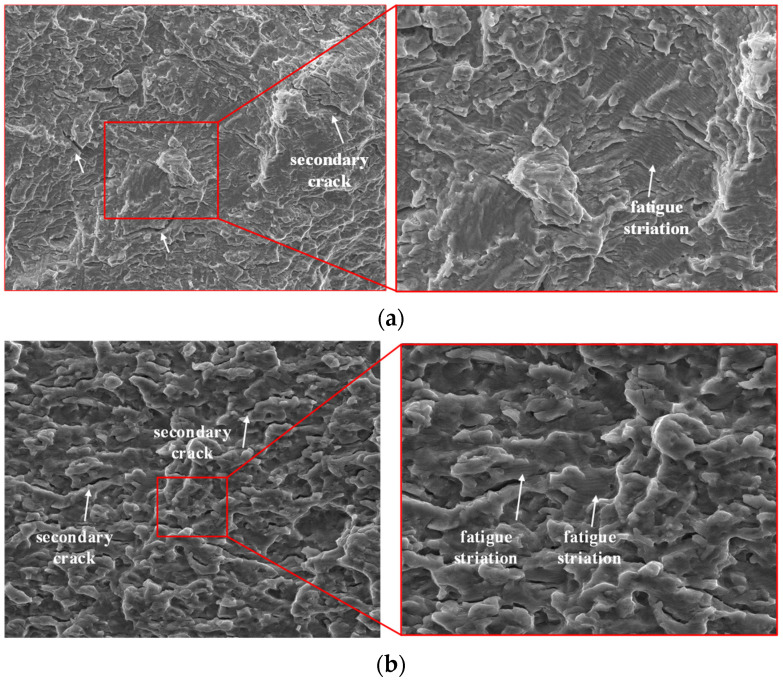
Fatigue crack propagation zone of the specimen. (**a**) Turned specimen; (**b**) Ultrasonic-impacted specimen.

Figure 14 shows the fatigue crack propagation area of the specimens. It can be seen from Figure 14a that the fatigue crack propagation area of turned specimen was a macroscopically quasi-cleavage fracture. The most typical feature of the microscopic morphology was the fatigue band. It can be seen that there existed a series of substantially parallel strips on the fatigue crack propagation zone, whose texture direction was perpendicular to the local crack propagation direction. Each fatigue strip basically corresponded to a cyclic load in the fatigue process, and the width of the fatigue strip was related to the crack growth rate. At about 1.05 mm from the fatigue source, the fatigue bandwidth was about 0.909–0.969 μm. Another notable feature of the fatigue propagation zone was more secondary cracks. In the figure, secondary cracks perpendicular to the extension direction of the main crack can be observed. Small steps appeared in the crack propagation process. This is because of the repeated opening and friction of the upper and lower surfaces of the crack near the fatigue source. Figure 14b shows the fatigue crack propagation zone of the ultrasonic-impacted specimen. At the position about 1.02 mm away from the fatigue source, the fatigue bandwidth was about 0.714–0.803 μm, which was about 20% smaller than that of the turned specimen. It showed that in the same cycles, the crack propagation resistance of the ultrasonic-impacted specimen was bigger, and the crack growth rate was about 20% smaller than that of the turned specimen. This is due to the high density of dislocations formed in the impacted surface layer, which formed a barrier to crack growth and reduced the crack growth rate. At the same time, some small and relatively smooth surfaces appeared near the fatigue source, which indicated that when the crack tip opened and closed, the surface roughness was reduced by repeated friction. This also indirectly indicated that the crack growth rate of the ultrasonic-impacted specimen was low in the early stage of crack growth, and the ultrasonic impact process significantly reduced the growth rate of fatigue cracks.

**Figure 15:**
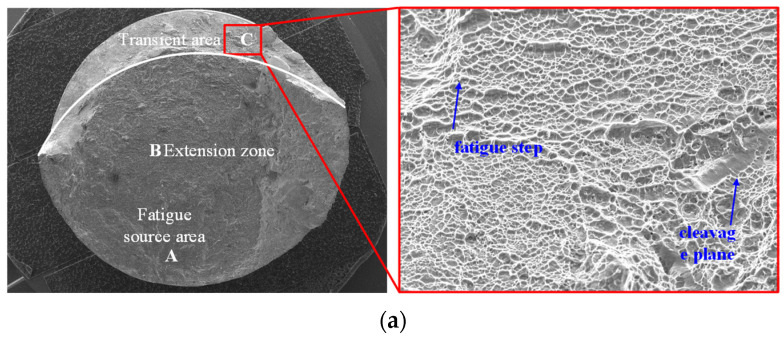

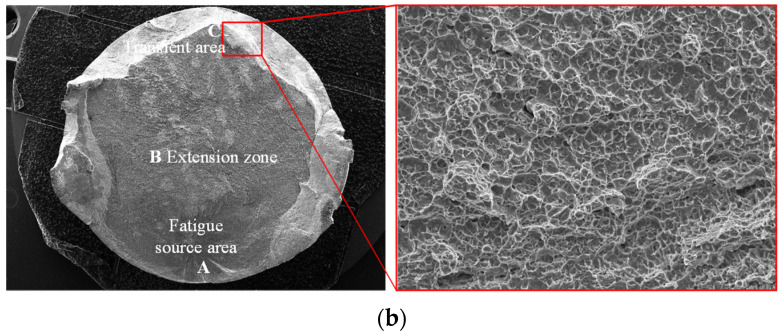
Fatigue transient area of turned and ultrasonic-impacted specimens. (**a**) Turned specimen; (**b**) Ultrasonic-impacted specimen.

Figure 15 is the transient area of the fatigue specimen. Figure 15a is the turned specimen, and Figure 15b is the ultrasonic-impacted specimen. It can be seen that the transient area of Ti-17 titanium alloy belongs to ductile fracture. This kind of fracture surface is relatively rough and has a large number of interconnected dimples of different sizes [26]. In the rapid propagation process of cracks, the ligament between the micropores in the direction of crack propagation and the crack tip became thinner and finally broke, which accelerated the fatigue crack propagation and formed dimples. Comparing the turned and ultrasonic-impacted specimens, in the transient area of the rapid crack propagation stage, the crack tip exceeded the impact area of the compressive stress field, grain refinement, and high-density dislocations introduced by ultrasonic impact strengthening. The inhibitory effect of ultrasonic impact on crack propagation almost disappeared. In general, compared with the turning process, ultrasonic impact strengthening prolongs the fatigue life of Ti-17 titanium alloy mainly by delaying the crack initiation stage and the initial stage of crack propagation.

## 4. Conclusions

(1) The fatigue life of ultrasonic-impacted specimens was increased by 73.9% compared with that of turned specimens. With the continuous increase in the fatigue cycle, the surface roughness of turned and ultrasonic-impacted specimens was also increased. The effect of ultrasonic impact mainly slowed down the appearance of surface slip bands at the initial stage of fatigue source formation.
(2) The residual stress in turned and ultrasonic-impacted specimens were both compressive residual stress states. The compressive residual stress and its affected layer depth continued to decrease with the increase of the fatigue cycle. The residual stress and its affected layer depth of the ultrasonic-impacted specimen were rapidly reduced by about 50% near the fracture stage.
(3) In the fatigue cycle, the microhardness of the turned specimens appeared to be a cyclic hardening phenomenon at the beginning of fatigue. At the same time, localized hardening occurred in the region. of 20–40 μm from the surface. Local hardened area occurred at 30–50 μm from the surface in ultrasonic-impacted samples. The surface hardness decreased rapidly near the fracture stage. The microhardness of these two kinds of specimens decreased with the increase of fatigue cycles, and the depth of the hardened layer did not change significantly.
(4) With the increase of the fatigue cycle numbers, the spacing of the fibrous gradually increased, and some scattered long strips of α phase appeared. This disintegrated the fibrous structural layer in the ultrasonic-impacted surface, while the plastic deformation layer depth decreased significantly. Compared with the turned surface layer, the attenuation process of the significant plastic deformation and the grain refinement in the ultrasonic-impacted surface layer need a longer period of time.
(5) The fatigue sources of the turned and ultrasonic-impacted specimens in this research were both located in the sub-surface. The fatigue source of the turned specimen and ultrasonic-impacted specimen was in the position of about 320 μm and 610 μm from the surface, respectively. The width of the fatigue strips of the ultrasonic-impacted specimen was 20% narrower than that of the turned specimen. In general, ultrasonic impact strengthening prolongs the fatigue life of Ti-17 mainly by delaying crack initiation and reducing the propagation speed in the initial stage of crack propagation.

In this paper, the changing law of surface integrity characteristics in the fatigue process has been studied relatively completely. The establishment of an accurate prediction model based on the surface integrity state and fatigue parameters will be a further research direction in the future.

## Figures and Tables

**Table 1:** Chemical composition of Ti-17 [mass fraction, %].

Alloying Element							Impurities					
Element	*Al*	*Sn*	*Zr*	*Mo*	*Cr*	*Ti*	*Fe*	*C*	*N*	*H*	*O*	*Other*
Content	4.5–5.5	1.6–2.4	1.6–2.4	3.5–4.5	3.5–4.5	balance	0.03	0.05	0.05	0.0125	0.08–0.13	0.4

**Table 2:** Mechanical properties of Ti-17.

Tensile Strength *σ*_b_ (MPa)	Yield Strength *σ*_0.2_ (MPa)	Elongation *δ* (%)	Shrinkage *Ψ* (%)
1219	1166	10.0	18.4

## Data Availability

The raw/processed data required to reproduce these findings cannot be shared at this time as the data also forms part of an ongoing study. This article does not contain any studies with human participants performed by any of the authors.

## References

[1] Tan L., Yao C., Zhang D., Ren J., Zhou Z., Zhang J. (2020). Evolution of Surface Integrity and Fatigue Properties after Milling, Polishing, and Shot Peening of TC17 Alloy Blades. Int. J. Fatigue.

[2] Mangardich D., Abrari F., Fawaz Z. (2019). A Fracture Mechanics Based Approach for the Fretting Fatigue of Aircraft Engine Fan Dovetail Attachments. Int. J. Fatigue.

[3] Elsheikh A.H., Shanmugan S., Muthuramalingam T., Thakur A.K., Essa F.A., Ibrahim A.M.M., Mosleh A.O. (2022). A Comprehensive Review on Residual Stresses in Turning. Adv. Manuf..

[4] Elsheikh A.H., Ashham M., Ali M.K.A., Rashad M., Haiou Z. (2019). Effect of Cutting Parameters on Surface Residual Stresses in Dry Turning of AISI 1035 Alloy. J. Braz. Soc. Mech. Sci. Eng..

[5] Hong J., Jiang L., Xu X., Ma Y. (2020). High Cycle Fatigue Failure with Radial Cracks in Gears of Aero-Engines. Chin. J. Aeronaut..

[6] Ye D. (2005). Investigation of Cyclic Deformation Behavior in the Surface Layer of 18Cr-8Ni Austenitic Stainless Steel Based on Vickers Microhardness Measurement. Mater. Chem. Phys..

[7] Haghshenas A., Khonsari M.M. (2018). Damage Accumulation and Crack Initiation Detection Based on the Evolution of Surface Roughness Parameters. Int. J. Fatigue.

[8] Martin U., Altenberger I., Scholtes B., Kremmer K., Oettel H. (1998). Cyclic Deformation and near Surface Microstructures of Normalized Shot Peened Steel SAE 1045. Mater. Sci. Eng. A.

[9] Han S., Lee T., Shin B. (2002). Residual Stress Relaxation of Welded Steel Components under Cyclic Load. Steel Res..

[10] Zaroog O.S., Ali A., Sahari B.B., Zahari R. (2011). Modeling of Residual Stress Relaxation of Fatigue in 2024-T351 Aluminium Alloy. Int. J. Fatigue.

[11] Saalfeld S., Oevermann T., Niendorf T., Scholtes B. (2019). Consequences of Deep Rolling on the Fatigue Behavior of Steel SAE 1045 at High Loading Amplitudes. Int. J. Fatigue.

[12] Isa M.R., Zaroog O.S., Ali F.S. (2018). Relationship between Compressive Residual Stress Relaxation and Microhardness Reduction after Cyclic Loads on Shotpeened ASTM A516 Grade 70 Steel. Key Eng. Mater..

[13] Benedetti M., Fontanari V., Monelli B.D. (2010). Numerical Simulation of Residual Stress Relaxation in Shot Peened High-Strength Aluminum Alloys under Reverse Bending Fatigue. J. Eng. Mater. Technol. Trans. ASME.

[14] Sano Y., Akita K., Takeda K., Sumiya R., Tazawa T., Saito T., Narazaki C. (2011). Stability of Residual Stress Induced by Laser Peening under Cyclic Mechanical Loading. Int. J. Struct. Integr..

[15] Gill C.M., Fox N., Withers P.J. (2008). Shakedown of Deep Cold Rolling Residual Stresses in Titanium Alloys. J. Phys. D Appl. Phys..

[16] Nikitin I., Altenberger I. (2007). Comparison of the Fatigue Behavior and Residual Stress Stability of Laser-Shock Peened and Deep Rolled Austenitic Stainless Steel AISI 304 in the Temperature Range 25–600 °C. Mater. Sci. Eng. A.

[17] Yan Z., Wang D., Wang W., Zhou J., He X., Dong P., Zhang H., Sun L. (2018). Ratcheting Strain and Microstructure Evolution of AZ31B Magnesium Alloy under a Tensile-Tensile Cyclic Loading. Materials.

[18] Wang H., Jing H., Zhao L., Han Y., Lv X., Xu L. (2017). Uniaxial Ratcheting Behaviour of 304L Stainless Steel and ER308L Weld Joints. Mater. Sci. Eng. A.

[19] Jia Y.F., Liu Y.X., Huang J., Fu Y., Zhang X.C., Xin Y.C., Tu S.T., Mao M.D., Yang F. (2019). Fatigue-Induced Evolution of Nanograins and Residual Stress in the Nanostructured Surface Layer of Ti-6Al-4V. Mater. Sci. Eng. A.

[20] Mortezavi V., Haghshenas A., Khonsari M.M., Bollen B. (2016). Fatigue Analysis of Metals Using Damping Parameter. Int. J. Fatigue.

[21] Kumar J., Rao A.V., Kumar V. (2015). Simulation of Elevated Temperature Fatigue Damage Evolution Using the Finite Element Method for near Alpha Titanium Alloy. Fatigue Fract. Eng. Mater. Struct..

[22] Griffits A.A. (1921). The Phenomena of Rupture and Flow in Solids. Phil. Trans. R. Soc. Lond. A.

[23] Lopes J.P., Malcher L. (2017). Fatigue Life Estimates under Non-Proportional Loading through Continuum Damage Evolution Law. Theor. Appl. Fract. Mech..

[24] Erdogan F., Tuncel O., Paris P.C. (1962). An Experimental Investigation of the Crack Tip Stress Intensity Factors in Plates under Cylindrical Bending. J. Fluids Eng. Trans. ASME.

[25] Mughrabi H. (2009). Cyclic Slip Irreversibilities and the Evolution of Fatigue Damage. Metall. Mater. Trans. B.

[26] Suresh S. (1998). Fatigue of Materials.

